# Scarcity of resources and inequity in access are frequently reported ethical issues for physiotherapists internationally: an observational study

**DOI:** 10.1186/s12910-021-00663-x

**Published:** 2021-07-20

**Authors:** Caroline Fryer, Andrea Sturm, Roswith Roth, Ian Edwards

**Affiliations:** 1 UniSA Allied Health and Human Performance, University of South Australia, GPO Box 2471, Adelaide, SA 5001 Australia; 2 Interuniversity College for Health and Development Graz, Castle of Seggau, Seggauberg, Austria; 3 University of Graz, Universitaetsplatz 2/III, 8010, Graz, Austria

**Keywords:** Physiotherapy, Ethics, professional, Survey

## Abstract

**Background:**

Little is known about the ethical situations which physiotherapists encounter internationally. This lack of knowledge impedes the ability of the profession to prepare and support physiotherapists in all world regions in their ethical practice. The purpose of the study was to answer the following research questions: What types of ethical issues are experienced by physiotherapists internationally? How frequently are ethical issues experienced by physiotherapists internationally? Can the frequency and type of ethical issue experienced by physiotherapists be predicted by sociodemographic, educational or vocational variables?

**Methods:**

An observational study was conducted in English using an online survey from October 2018 to May 2019. Participants were 1212 physiotherapists and physiotherapy students located internationally which represented less than 1% of estimated number of physiotherapists worldwide at that time. The survey questionnaire contained 13 items requesting demographic detail and knowledge of ethical codes and decision-making, and 46 items asking what frequency participants experienced specific ethical issues in four categories: (A) Physiotherapist and patient interaction (19 items), (B) Physiotherapist and other health professionals including other physiotherapists (10 items), (C) Physiotherapists and the system (5 items) and (D) Professional and economic ethical situations (12 items).

**Results:**

The two most frequently experienced ethical issues were ‘Scarce resources and time affecting quality of physiotherapy treatment’ and ‘Physiotherapy not accessible to all people in society who need it’. These items were experienced, on average, more often than monthly. Interprofessional practice also presented frequent ethical issues for participants. Ethical issues related to the context of ‘Physiotherapists and the system’ were most frequently experienced for all world regions. Working longer years in physiotherapy and learning about ethics in basic physiotherapy education was associated with participants reporting lower frequencies of ethical issues across all contexts.

**Conclusion:**

This study provides the first global profile of ethical issues experienced by physiotherapists. Societal and cultural systems are key influences on physiotherapists’ ethical practice. Physiotherapists globally need support from their work organisations, academic institutions and professional associations, and robust ethical training, to assist them to be active moral agents in their practice.

**Supplementary Information:**

The online version contains supplementary material available at 10.1186/s12910-021-00663-x.

## Background

Little is known about the ethical situations which physiotherapists encounter internationally. There is evidence that physiotherapists in a wide variety of practice contexts face ethical issues, based on published research from mostly Western societies [1–9]. A small number of studies are being reported from culturally different areas of the world [10–14], but no previous study has determined the scope and nature of ethical issues in the physiotherapy profession worldwide. What we do know from existing research is that ethical issues are part of everyday physiotherapy practice across fields of the physiotherapy profession, causing moral distress for practitioners and affecting quality and outcomes of care [1, 2, 4, 9]. This part of professional practice cannot be ignored, rather it needs to be understood to be addressed appropriately and effectively. Lack of knowledge impedes the ability of the profession to prepare and support physiotherapists in all world regions in their ethical practice.

Initial investigations into ethical issues for physiotherapists were conducted in the 1980s and 1990s in America and the United Kingdom [1, 4, 6]. Two published surveys of physiotherapist practitioners at this time found the ethical issues they frequently encountered in practice were decision-making about who to treat, managing the expectations of patient and families about treatment, economic resource constraints on practice, and conflict in interprofessional relationships [1, 4]. A small panel of American physiotherapy experts identified further important ethical issues for the profession included appropriate clinical competence, achieving informed consent and maintaining confidentiality, product endorsement, truth in advertising, overutilization of services and sexual misconduct by physiotherapists [6]. Over the last 20 years, studies from Canada, America, Europe and Australia have continued to report, and expand, on these themes with increased identification of issues of patient autonomy, multiple stakeholders and conflicts of interest in decision-making, diversity in patients’ cultural values and beliefs, business and productivity conflicts with patient-centred care, professional boundaries, and the physiotherapist’s role as advocate [2, 3, 5, 7–9].

Recent publications provide initial insights into the ethical experience of physiotherapists in non-Western societies [10–12, 14]. An African perspective in studies from Ghana and Zambia report physiotherapists frequently experience issues with gift-giving and professional boundaries in the patient-therapist relationship [11, 14]. Physiotherapists in Zambia also identified conflicts between culture and the treatment process, issues of patient safety with home exercise programs, interprofessional conflict and competency of informed consent [11]. In Ghana, providing physiotherapy care when resources are limited was the most frequent ethical issue, followed by managing patient and family expectations [14]. Physiotherapists providing end of life care in another African country, Nigeria, identified ethical issues of conflict between cultural beliefs and patient autonomy when disclosing information about dying. Other key issues were late referrals affecting quality of care, managing patient and family expectations, and determining treatment effectiveness [10]. Comparatively, physiotherapists providing end of life care in Brazil reported key ethical conflicts were providing therapeutic treatment when there is non-acceptance of death, and providing humanistic patient care when it exposes the physiotherapist to the potential harm of emotional distress [15]. Physiotherapists in Iran report a different profile of ethical issues in practice including physiotherapists acting in self-interest over patient-interest and physiotherapists acting on personal rather than professional beliefs in the absence of ethics training, as well as issues of affordability of care, patient autonomy, and maintaining privacy [12]. This identification of new issues and contextual differences in previously-known issues from the emerging research demonstrates there is much more to be known about ethical challenges in international physiotherapy practice.

An integral guide to ethical and professional physiotherapy practice is a code of ethics. Practitioners of member countries of the World Physiotherapy organisation are expected to comply with a code of ethics based on principles of respect for individual autonomy, honesty, equity and justice [16]. As the profession develops, an understanding is needed of how physiotherapists translate the code of ethics to the many diverse political and cultural contexts of clinical practice across world regions. A study of ethical issues experienced by physiotherapists internationally can inform which ethical obligations are challenged in everyday practice. This knowledge can then be used by the global profession to be culturally responsive in how it guides physiotherapists to be moral agents and active participants in improvement and integrity of health care provision in the countries in which they practice.

This paper reports findings from the ESPI-Study (Ethical Situations in Physiotherapy Internationally), which aimed to describe the ethical landscapes for physiotherapists internationally. The aim of the research reported in this paper was to understand the context of ethical practice for the international physiotherapy profession. The research questions were:

1. What types of ethical issues are experienced by physiotherapists internationally?
2. How frequently are ethical issues experienced by physiotherapists internationally?
3. Can the frequency and type of ethical issue experienced by physiotherapists be predicted by sociodemographic, educational or vocational variables?

## Method

### Design

A questionnaire was used to examine what ethical issues are most frequently experienced by physiotherapists internationally. The list of ethical issues included in the questionnaire was expanded from previous surveys to include issues reported in contemporary literature [1, 3, 4, 6–8, 10, 11, 17]. The questionnaire was developed in the English language with FleshKincaid English readability level 6.7. A pilot survey was conducted with eight physiotherapists including five physiotherapists for whom English was not their first language. From the pilot feedback, minor amendments were made to the questionnaire wording, and the use of examples and a possibility to use a dictionary was added to the questionnaire introduction section. An ethical situation was defined for participants as ‘any issue in which an ethical tension is created in the physiotherapist’s practice—for example, a conflict of values, beliefs, or norms; uncertainty as to the appropriate ethical action to take; or distress arising from an inability to act in a way that met the professional’s (or the profession’s) ethical standards’.

The final survey was conducted online from October 2018 to May 2019 using SurveyMonkey software (accessed on 11th April 2018 for the first time). The first page of the survey provided participant information about the study. If the participant consented to participate, they could continue the survey. If they did not consent to participate, they could exit the survey at that point. This study was approved by the University of South Australia's Human Research Ethics Committee and the Institute of Ethics and Right in Medicine of the University of Vienna.

The questionnaire contained 60 items in three sections (Additional file 1: Appendix 1). Section 1 contained 13 items requesting participant age, gender, nationality, level of physiotherapy education, type of workplace, location of workplace, paying sources and field of physiotherapy practice. Two items asked participants their knowledge of ethical codes and decision-making. Section 2 contained 46 items asking what frequency participants experienced specific ethical issues: ‘daily (= 1)’, ‘weekly (= 2)’, ‘monthly (= 3)’, ‘yearly or less (= 4)’, or ‘never (= 5)’. The specific ethical issues were presented in four categories based on context: (A) Physiotherapist and patient interaction (19 items), (B) Physiotherapist and other health professionals including other physiotherapists (10 items), (C) Physiotherapists and the system (5 items) and (D) Professional and economic ethical situations (12 items). Section 2 categories and items were presented in random order. Section 3 asked participants to describe an ethical situation they had experienced which was not mentioned in the questionnaire’s list of items, if this applied to them. Results from ﻿﻿﻿Sects.﻿﻿ ﻿1 and 2 are reported in this paper.

### Participants

The target group for the survey was physiotherapists and physiotherapy students located internationally with access to the internet and English proficiency to complete the questionnaire. The survey was not timed. Participants were recruited using purposive and snowball sampling. The survey URL link was distributed internationally in English in four ways. Firstly, a licence-free advertisement was shared on physiotherapy professional social media networks identified by the authors (Twitter, Facebook, LinkedIn, ResearchGate). Secondly, a paid pop-up advert was featured over a period of eight weeks from 10th October until 30th November 2018 on the homepage of Physiopedia, a freely-accessible online physiotherapy database and the survey was promoted in their online-journal ‘Physiospot’. Thirdly, an invitation to participate was emailed to all national World Confederation for Physical Therapy (WCPT) associations (now titled ‘World Physiotherapy’) with a request to forward the link to members. Finally, 300 printed invitations were handed out at the 2019 WCPT congress.

### Data analysis

Sociodemographic characteristics of participants were descriptively analysed. Some participants did not answer all items and the respective number of respondents for each item is presented in tables. A drop-out analysis evaluated the sociodemographic and occupational differences between participants who finished all three sections of the questionnaire and those who dropped out earlier.

Mean frequencies for each item were calculated using the values from daily (= 1) to never (= 5); the more often the item was experienced, the lower the score. The normal or equal distribution of item responses in Sect. 2 were evaluated by Kolmogorov–Smirnoff and Chi^2^ tests respectively. Neither a normal or equal distribution was found for any item. The item analyses of the four respective categories showed good reliabilities (Cronbach α 0.77–0.91, item-scale correlation 0.39–0.67). The four categories were consequently treated as scales with means and standard deviations (SD). Forward stepwise multiple regression analyses were calculated for each scale as criteria (including predictors *p* 0.05, excluding predictors *p* 0.10) with biographical predictors (gender, WCPT membership, WCPT region), vocational variables (years of working, types and number of workplaces, working areas, working fields, and paying sources) and educational variables (education in physiotherapy vs. other degrees, learned about codes of conduct/ethics, learned about ethical reasoning/decision-making). Because the correlation between age and working years was high (r = 0.0.915, *p* < 0.001), working years was included as a predictor and not age. Dummy variables were used for nominal variables. A description of all dummy variables is given in Additional file 2: Appendix 2.

## Results

### Participant demographics

1,212 individuals participated in the online questionnaire. This response rate represents less than 1% of the estimated 1,583,361 physiotherapists worldwide in 2018 [18]. It is not known how many international physiotherapists were reached by study recruitment methods, or had internet access or a level of English proficiency to access the online questionnaire, to determine a more precise study cohort. Participant age ranged from 18 to 76 years (Table 1) and most were female (67%, male 32%, other or diverse 1%). Participants were from 94 different countries. Table 1 presents the distribution of the sample according to gender and WCPT region. Participants had worked in physiotherapy for a mean of 13.5 years (SD 11.0). There were 264 participants (22%) who indicated they were currently undertaking some form of physiotherapy training. Many participants reported working in more than one type of workplace currently or over their career (mean 2.6, SD 1.7). The mean number of physiotherapy fields participants had practiced in was 5.1 (SD 3.4). Table 2 presents the education and occupation characteristics of the sample by gender.

**Table 1:** Number (%) of participants for each geographic region by gender

Geographic region	All n = 1212	Female n = 815	Male n = 389	Diverse n = 8
Africa region	141 (12)	79 (7)	62 (5)	0 (0)
Asia Western Pacific region	383 (32)	259 (21)	121 (10)	3 (0)
Europe region	534 (44)	377 (31)	154 (13)	3 (0)
North America Caribbean region	139 (11)	92 (8)	45 (4)	2 (0)
South America region	15 (1)	7 (1)	7 (1)	0 (0)

**Table 2:** Mean age (SD), and number (%) of participants for each occupation and education characteristic by gender

Characteristic	All n = 1212	Female n = 815	Male n = 389	Diverse n = 8
Age (years)	35.3 (11.8)	35.4 (12.2)	34.9 (10.7)	38.1 (14.8)
Type of workplace				
Private	1103 (91)	727 (89)	373 (96)	3 (38)
Government/public	891 (74)	632 (78)	256 (66)	3 (38)
Teaching institution	276 (23)	185 (23)	90 (23)	1 (13)
Research institution	96 (8)	67 (8)	29 (7)	0
Sports club	142 (12)	86 (11)	55 (14)	1 (13)
Self employed/owner	455 (38)	278 (34)	174 (45)	3 (38)
Other	140 (12)	107 (13)	33 (8)	0
Area where workplace located				
Rural area	120 (10)	79 (10)	38 (10)	3 (38)
Urban area	742 (61)	496 (61)	242 (62)	4 (50)
Both areas	346 (29)	237 (29)	108 (28)	1 (13)
Paying sources				
Private funding (patient or family)	682 (56)	440 (54)	236 (61)	6 (75)
Private funding (organization)	557 (46)	375 (46)	178 (46)	4 (50)
Public/governmental funding	549 (45)	371 (46)	175 (45)	3 (38)
Combination of public/governmental and private	609 (50)	410 (50)	196 (50)	3 (38)
Charities	153 (13)	106 (13)	46 (12)	1 (13)
Other	30 (2)	22 (3)	8 (2)	0
Field of physiotherapy practice				
Acupuncture, dry needling	170 (14)	102 (13)	66 (17)	2 (25)
Animal	13 (1)	11 (1)	0	2 (25)
Aquatic	178 (15)	133 (16)	44 (11)	1 (13)
Cardiorespiratory	376 (31)	254 (31)	120 (31)	2 (25)
Education	349 (29)	237 (29)	108 (28)	4 (50)
Disability	289 (24)	207 (25)	81 (21)	1 (13)
Health promotion	300 (25)	208 (26)	89 (23)	3 (38)
Information management	38 (3)	19 (2)	18 (5)	1 (13)
Management/administration	194 (16)	132 (16)	61 (16)	1 (13)
Mental health	97 (8)	70 (9)	26 (7)	1 (13)
Neurology	493 (41)	332 (41)	158 (41)	3 (38)
Occupational Health/ergonomics	173 (14)	109 (13)	61 (16)	3 (38)
Oncology/palliative care	163 (13)	120 (15)	42 (11)	1 (13)
Orthopaedics/manual therapy	737 (61)	472 (58)	261 (67)	4 (50)
Older people	520 (43)	357 (44)	160 (41)	3 (38)
Paediatrics	339 (28)	244 (30)	93 (24)	2 (50)
Rehabilitation	724 (60)	470 (58)	249 (64)	5 (63)
Research	216 (18)	136 (17)	78 (20)	2 (50)
Sport	419 (35)	242 (30)	173 (44)	4 (50)
Women’s, men’s and pelvic health	215 (18)	170 (21)	44 (11)	1 (13)
Other	126 (10)	89 (11)	36 (9)	0
Highest educational level achieved (in physiotherapy or other discipline)				
Bachelor/diploma	510 (42)	357 (44)	150 (39)	3 (38)
Graduate diploma	110 (9)	68 (8)	42 (11)	0
Masters degree	310 (26)	193 (24)	117 (30)	0
Professional doctorate	105 (9)	71 (9)	31 (8)	3 (38)
Research doctorate	59 (5)	39 (5)	18 (5)	2 (50)
Other	115 (9)	84 (10)	31 (8)	0
Learned about code of conduct/ethics during basic physiotherapy education				
Yes	893 (74)	591 (73)	298 (77)	4 (50)
No	216 (18)	156 (19)	61 (16)	1 (13)
Don’t know	99 (8)	68 (8)	29 (7)	2 (25)
Learned about specific ethical decision-making/reasoning frameworks during basic physiotherapy education				
Yes	581 (48)	373 (46)	204 (52)	4 (50)
No	399 (33)	278 (34)	121 (31)	0
Don’t know	231 (19)	162 (20)	63 (16)	4 (50)

### Flow of participants through the study

846 participants (69.8% of total) completed the survey. Drop-outs were significantly younger (mean age 31.35 years) than participants who completed all Sections (36.95 years) [F_1,1209_ = 60.442, *p* < 0.001] and more participants who reported being in training dropped out (Chi^2^ = 89.11, *p* < 0.001). When statistically comparing the four survey scales, the only significant difference between physiotherapists in training and working physiotherapists was in scale B ‘Physiotherapist and other health professionals including other physiotherapists’. Participants in training reported experiencing the issues listed in questions 33, 34, and 46 (Additional file 1: Appendix 1) significantly less frequently than working physiotherapists. The rates of drop-out across regions differed by, at most, 16.8% (28.6% Africa region, 34.4% Asian Western Pacific region, 29.2% Europe Region, 23.2% North American Caribbean region, 40% South America region) [Chi^2^ = 7.875, *p* = 0.097]. Participants who dropped out had, on average, worked a shorter time in physiotherapy than participants who stayed in the study (mean 11.16 vs. 14.22 years) [F_1,945_ = 13.649, *p* < 0.001] and practiced in less fields of physiotherapy (mean 4.5 vs. 5.3, F_1,1206_ = 17.361, *p* < 0.001). More respondents than expected with a Bachelor/Diploma in physiotherapy and less than expected with a Masters degree in physiotherapy dropped out of the study (Chi^2^ = 20.685, *p* < 0.001). Characteristics of completers and non-completers of the survey are given in Additional file 3: Appendix 3.

### Type and frequency of experiencing ethical issues

The mean frequency that participants reported experiencing ethical issues are ranked from most frequent to least frequent for the total cohort in Table 3. The two most frequently experienced ethical issues were the only two items experienced, on average, more often than monthly. Most ethical issues were reported to be experienced between monthly and yearly (30/46 items). The nine most frequently experienced issues were from scale B ‘Physiotherapist and other health professionals including other physiotherapists’ (4 items), scale C ‘Physiotherapists and the system’ (3 items) and scale D ‘Professional and economic ethical situations’ (2 items). Ethical issues relating to scale A ‘Physiotherapist and patient interaction’ were not represented in the overall rankings until the tenth item in the ranking. Overall, the mean frequency of experiencing an ethical issue was similar across the four scales with all mean values more frequent than yearly (scale A mean 3.96, SD 1.01; scale B mean 3.58, SD 1.36; scale C mean 3.24, SD 1.65; scale D mean 3.66, SD 1.48). Scale C was most frequently experienced for all geographic regions (African mean 3.12, SD 0.84; Asia Western Pacific mean 3.29, SD 0.98; European mean 3.28, SD 0.92; North America Caribbean mean 3.12, SD 0.89; South American mean 2.78, SD 0.84).

**Table 3:** Ranking of mean frequency that ethical issues were experienced for total cohort and comparative rankings for WCPT regions (most frequent–least frequent)

Item	Scale*	N	Mean** (SD)	Total cohort ranking	Africa region ranking	Asia Western Pacific region ranking	Europe region ranking	North America Caribbean region ranking	South America region ranking
45. Scarce resources and time affecting quality of physical therapy treatment	C	857	2.39 (1.3)	1	1.5	2	1	2	1
56. Physical therapy not accessible to all people in society who need it, e.g. due to cost, lack of services in regions, or discrimination by health care system	D	847	2.64 (1.4)	2	1.5	1	2	1	5
40. Respecting the patient's therapeutic relationship with other health professionals, when the physical therapist disagrees with the other health professional's opinion	B	855	2.95 (1.2)	3	4	3	3	3.5	19.5
36. Miscommunication, or lack of communication, between physical therapists and other health professionals causing errors and affecting quality in patient care	B	855	3.10 (1.2)	4.5	7	4.5	4	7.5	8
39. Referrals, or absence of referrals, from other health professionals that constrain the quality of physical therapy services	B	855	3.10 (1.2)	4.5	3	4.5	5.5	7.5	10
52. Lack of evidence available to support effectiveness and safety of physical therapy practices	D	847	3.19 (1.3)	6	5	7	5.5	11	8
41. Conflict with another health professional about patient’s management	B	855	3.26 (1.1)	7	10	6	9	11	6
43. Physical therapist required by an organisation or system to discharge patient from treatment based on reasons other than patient’s clinical need, e.g. insurance limits, health care system policy	C	857	3.27 (1.3)	8	16	10	10	3.5	4
44. Physical therapist prevented by an organisation or system from treating patient based on clinical need, e.g. health insurance will not cover condition, health care system policy does not allow	C	857	3.29 (1.4)	9	13	8	11	5.5	11
17. Mismatch/discrepancy between patient’s or family/caregivers’ wishes and physical therapist’s professional judgement	A	844	3.31 (1.1)	10	11	11	7	9	16
24. A purposeful absence of truth-telling by patient during treatment	A	844	3.33 (1.1)	11	6	9	12	11	23
16. Mismatch/discrepancy between the patient’s expectations and the physical therapist’s expectations of the therapeutic relationship	A	844	3.37 (1.1)	12	15	13	8	14	23
57. Conflict in duties toward employer, third-party payer, and the patient	D	847	3.47 (1.2)	13	18	14	18	5.5	13.5
31. Continuing physical therapy treatment for patient’s psychological/psychosocial support after treatment goals have been reached	A	844	3.51 (1.1)	14	19	15	23	18	12
58. A lack of advocacy for patient’s interests, needs or supports when they are unable to advocate for themselves	D	847	3.55 (1.2)	15	12	12	24.5	13	13.5
47. Withholding or limiting physical therapy services to improve work conditions of the physical therapy provider or for the convenience of the physical therapist, e.g. time, location	C	856	3.57 (1.3)	16	20	16	14	21	8
37. Physical therapist aware of misconduct by other health professionals, e.g. incompetency, violating laws and professional obligations	B	855	3.58 (1.2)	17	8	19	16	24	27.5
38. Prescription and ongoing provision of analgesics and/or sedatives to patients without appropriate review over time	B	854	3.59 (1.3)	18	14	28	15	15	19.5
46. Physical therapist pressured by organisation or system to return patient to sport or work commitments too early	C	857	3.68 (1.2)	19	29.5	26	19	16	2
51. Inadequate/unlawful record keeping by physical therapist	D	847	3.69 (1.3)	21.5	9	29.5	21	17	17.5
14. Absence of shared decision-making between patient and physical therapist, e.g. paternalism, not culturally wanted or accepted	A	843	3.69 (1.1)	21.5	32	27	17	19	23
29. Physical therapist prioritising patients for treatment based on reasons other than patient’s clinical need, e.g. cherry picking of easier patients, likelihood of improvement, economic potential	A	843	3.72 (1.2)	23.5	26	24	22	22.5	30.5
53. Physical therapist practicing outside of personal scope of knowledge and skills	D	847	3.72 (1.2)	23.5	23	20	20	39	39.5
54. Physical therapist overtreating patients for own economic gain	D	847	3.73 (1.3)	24	24	25	24.5	26	30.5
34. Bullying or harassment of physical therapist by other health professional(s)	B	855	3.79 (1.2)	25	22	18	31	27.5	19.5
55. Conflict between physical therapist’s professional obligations (as per code of ethics) and cultural or personal values	D	847	3.80 (1.2)	26.5	21	18	30	27.5	34
18. Patient’s privacy and/or dignity not respected during physical therapy treatment, e.g. not draping appropriately, gossiping about patients	A	843	3.80 (1.1)	26.5	28	33	23	22.5	30.5
28. Stopping treatment of a patient when they did not comply with physical therapist’s instruction or advice	A	844	3.83 (1.0)	28.5	35	32	26.5	20	16
30. Concerns of the physical therapist regarding treatment of terminally ill patients, e.g. deciding benefit or harm to the patient, futility of treatment, lack of resources	A	844	3.83 (1.2)	28.5	27	22	28	34.5	25.5
15. Failure to gain informed consent, e.g. cultural differences, cognitive impairment, not attempted	A	844	3.84 (1.1)	30	25	17	26.5	37.5	39.5
59. Physical therapist recommending and selling products for own economic gain	D	847	3.90 (1.2)	31	17	29.5	36	37.5	16
48. Breach of patient confidentiality by physical therapist	D	847	3.94 (1.1)	32	33	31	34	29.5	43
26. An absence of purposeful truthtelling by therapist during treatment	A	843	3.97 (1.1)	33.5	34	34	29	34.5	34
42. Inappropriate, insulting or offending behavior among colleagues on social media	B	855	3.97 (1.2)	33.5	31	36	35	25	19.5
33. Other health professionals seeking financial or other benefit from referring patients to physical therapists	B	855	4.02 (1.3)	35	38	23	41	31	27.5
50. Overcharging patients for physical therapy services	D	847	4.06 (1.2)	36	29.5	37	39	29.5	3
25. Violence or threatening behaviour by patient towards physical therapist	A	844	4.07 (1.0)	37	39	35	37	36	25.5
22. Discrimination by physical therapist towards patient on basis of age, gender, appearance, culture or religion, e.g. refusal to treat, lack of respect for cultural beliefs, poor quality treatment	A	844	4.23 (1.0)	38	40	41	38	40	43
49. Fraudulent billing for physical therapy services	C	847	4.24 (1.1)	39	37	38.5	43	33	41
23. Physical therapist accepting inappropriate gifts or gratuities	A	844	4.27 (1.0)	40	36	38.5	42	42	30.5
21. Inappropriate relationship between patient and physical therapist during treatment, e.g. intimate friendship, business partnership	A	844	4.28 (1.0)	41	41.5	40	40	43	34
19. Sexual harassment by patient during treatment	A	844	4.32 (0.8)	42	44	43.5	32	32	43
32. Physical therapist abusing their status/power to influence patient’s behavior for their own interest	A	844	4.38 (1.0)	43	43	43.5	44	41	37.5
35. Violence or threatening behaviour against patients by other health professionals	B	855	4.39 (0.9)	44	41.5	42	45	44	36
27. Violence or threatening behaviour by physical therapist towards patient	A	844	4.72 (0.7)	45	45	45	33	46	45.5
20. Sexual harassment by physical therapist during treatment	A	844	4.81 (0.5)	46	46	46	46	45	45.5

*Scale A = Physical Therapist and Patient Interaction, B = Physical Therapist and other Health Professionals (including other Physical Therapists), C = Physical Therapist and the System, D = Professional and Economic Ethical Situations

**Experienced daily = 1, weekly = 2, monthly = 3, yearly or less = 4, never = 5

The comparative rankings between WCPT regions of the mean frequency of experiencing each ethical issue is shown in Table 3. Survey item 45 ‘Scarce resources and time affecting quality of physiotherapy treatment’ was the first or second ranked most frequently experienced issue for all WCPT regions. The five most frequently experienced ethical issues for the total cohort were all ranked within the ten most frequently experienced issues for each WCPT region.

### Predicting frequency of experiencing ethical issues

Experiencing ethical issues in scale A could be significantly predicted (R = 0.269, R^2^ = 0.072, F_6,707_ = 9.175, *p* < 0.001) with six predictor variables explaining 7.2% of the variance. Participants who were female or male, worked in fewer physiotherapy fields, worked at more types of workplaces, worked longer years as a physiotherapist and learned about ethic codes during basic education experienced ethical issues less frequently in this scale. Experiencing ethical issues in scale B could be significantly predicted (R = 0.306, R^2^ = 0.094, F_6,723_ = 12.465, *p* < 0.001) with six predictors explaining 8.6% of the variance. Participants who were female, worked longer as a physiotherapist, and learned about ethic codes during basic education reported experiencing ethical issues less frequently in this scale. Being gender diverse, working in more fields of practice and coming from the Africa region was predictive of experiencing ethical issues more frequently for scale B. Experiencing ethical issues in scale C could be significantly predicted (R = 0.182, R^2^ = 0.033, F4,721 = 6.187, *p* < 0.001) with four predictors explaining 3.3% of the variance. The longer the physiotherapist had worked predicted less frequently experiencing ethical issues for this scale. The more fields of physiotherapy practice, working in a rural area and not learning about ethic codes in basic education predicted more frequently experiencing issues in scale C. Experiencing ethical issues in scale D could be significantly predicted (R = 0.279, R^2^ = 0.078, F_6,711_ = 9.982, *p* < 0.001) with six predictors explaining 7.8% of the variance. Being female, working more years, coming from the European region and having learned about ethics codes in basic education predicted less frequently experiencing ethical issues in scale D. Working in more physiotherapy fields and coming from the Africa region predicted more frequently experiencing issues in this scale (see Table 4).

**Table 4:** Prediction of the four category scales* (significant predictors only)

Predictors	Criteria											
	Scale A			Scale B			Scale C			Scale D		
	β	T	*p*	β	T	*p*	β	T	*p*	β	T	*p*
Gender												
Female	0.65	2.44	0.01	0.88	2.41	0.01	–	–	–	0.11	3.00	0.00
Male	0.55	2.06	0.04	–	–	–	–	–	–	–	–	–
Diverse	–	–	–	− 0.84	− 2.34	0.02	–	–	–	–	–	–
WCPT regions												
Africa	–	–	–	–	–	0.01	–	–	–	–	–	0.01
Asia & Western Pacific	–	–	–	0.10	2.84	–	–	–	–	0.96	2.46	–
Europe	–	–	–	–	–	–	–	–	–	–	–	0.00
North America & Caribbean	–	–	–	–	–	–	–	–	–	0.13	3.18	
South America	–	–	–	–	–	–	–	–	–	–	–	–
Working years	0.11	3.00	0.00	0.19	5.07	< 0.00	0.14	3.71	< 0.00	0.10	2.45	0.02
Areas												
Rural	–	–	–	–	–	–	–	–	0.05	–	–	–
Urban	–	–	–	–	–	–	0.07	2.01	–	–	–	–
Both	–	–	–	–	–	–	–	–	–	–	–	–
Number of type of workplaces	0.84	2.06	0.39	–	–	–	–	–	–	–	–	–
Number of working fields	− 0.11	− 3.63	< 0.00	− 0.16	− 4.31	< 0.00	− 0.10	− 2.55	0.01	− 0.11	− 2.97	0.00
Learned code of conduct												
Yes	0.1y	4.24	< 0.00	0.13	3.59	< 0.00	–	–	–	0.14	3.68	< 0.00
No	–	–	–	–	–	–	0.09	2.30	0.02	–	–	–
Don’t know	–	–	–	–	–	–	–	–	–	–	–	–
	F_6.707_ = 9.18, *p* < 0.001			F_6,723_ = 12.47, *p* < 0.001			F_4,721_ = 6.19, *p* < 0.001			F_6,711_ = 9.98, *p* < 0.001		
	R = 0.27, R^2^ = 0.07 R^2^corr = 0.064			R = 0.31, R^2^ = 0.09 R^2^corr = 0.086			R = 0.18, R^2^ = 0.03 R^2^corr = 0.028			R = 0.28, R^2^ = 0.08 R^2^corr = 0.070		

*Scale A = Physical Therapist and Patient Interaction, B = Physical Therapist and other Health Professionals (including other Physical Therapists), C = Physical Therapist and the System, D = Professional and Economic Ethical Situations. Only significant predictors are shown; variables of paying sources, WCPT membership, degree in physiotherapy vs. other disciplines, learning about ethic decision making were not significant predictors in any scale

## Discussion

The reported study is the first to attempt to establish an international profile of ethical issues experienced by physiotherapists in their everyday practice. The study found physiotherapists in all world regions are most frequently being challenged by societal and organisational systems limiting access to physiotherapy care or the resources needed to provide equitable care. This same issue of ‘scarce resources and time affecting quality of treatment’ was being raised as a key ethical issue by physiotherapists in America and the United Kingdom over thirty years ago [1, 4]. The current study finding may reflect the introduction of Western-based models of health care in more countries influencing resources available for physiotherapy. It may also reflect the lack of research on ethical issues from non-western countries preventing earlier identification in other regions. The ubiquity of this ethical issue is concerning as the estimated need for rehabilitation globally is one in every three people during illness or injury, with musculoskeletal disorders presenting the greatest rehabilitation need for children and adults [19]. Access to early physiotherapy intervention is a key way to improve function and independence for people thereby reducing disability and its associated costs for the individual and their community [20–22]. The high ranking of issues relating to external constraints on physiotherapy quality and access situates the international physiotherapy profession in the predicted ‘period of social identity’ [23] and is consistent with recent calls for greater recognition of the societal realm in codes of ethics [2, 24]. The persistence of this ethical issue over many years for physiotherapists in some countries points to the difficulty experienced by the profession in addressing it. Previous recommendations have focused on responses by the individual physiotherapist, yet a recent study from the United States identified social responsibility as a professional value that physiotherapists can lack awareness of, or have difficulty integrating into, their practice [25]. It has also been suggested that physiotherapists may not perceive themselves capable of moral agency in justice at a societal level [26]. This implies that the contemporary and international profession needs to strengthen both the capacity of the individual physiotherapist and the capacity of physiotherapy organisations and associations to achieve change in this ethical situation. For example, building the skill set of physiotherapy organisations to advocate and campaign at different levels of government for changes in health policy that impact equitable delivery of physiotherapy care. Or facilitating professional networks for individual physiotherapists to join with other individuals in sharing experiences and petitioning for change in local organisational practices.

The finding that a lack of training in ethics codes was associated with more frequently experiencing ethical issues involving system constraints points to the importance of a strong ethics curriculum in the training of twenty-first century physiotherapy graduates. There is a small body of work that has investigated effective ways to teach ethics curriculum in physiotherapy training [27–32]. They report teaching approaches that engage students in critical thinking and decision-making about ethical issues beyond knowledge and application of normative principles. Contemporary teaching approaches will need to incorporate a relevant skillset for physiotherapists to act for change in organisational and societal contexts [9]. According to a Population-Based Practice framework for achieving change in policies and laws, such skills include consultation, collaboration, advocacy and policy development [33]. In addition, individual physiotherapists will need the support of professional bodies to strengthen their capability to influence social policy and health care reforms.

Difficulties in relationships with other health professionals is causing ethical issues at least monthly for physiotherapists globally. Interprofessional practice is widely accepted to be desirable in health care to benefit health care outcomes and as a workforce imperative [34], yet this study adds to previous evidence of relational challenges affecting care [1, 2, 7, 11]. The interprofessional context of ethical issues was more frequently experienced by participants from the Africa region where physiotherapists practice within a paternalistic model and physiotherapy referrals can be delayed by other health practitioners [10, 11]. This again highlights the need for ethical issues to be addressed by broader system change. Respect and collaborative relationships between health professionals are key factors for successful interprofessional practice [35] and the recent international emphasis on interprofessional training in undergraduate curricula [34] as well as the profession’s involvement in a global campaign supporting professional recognition in positive practice environments [36, 37] may influence a stronger understanding of other disciplines and culture of respect between professionals in workplaces.

Working longer years in physiotherapy and learning about ethics in basic physiotherapy education was associated with participants reporting lower frequencies of ethical issues across all contexts. An explanation for this finding is not directly apparent, and likely to be complex. It does suggest some kind of cognitive filtering process is occurring where ethical issues identified in practice by less-experienced physiotherapists are being viewed differently by more-experienced physiotherapists. This may be an example of longer exposure to organisational pressures leading to individuals having to accommodate and rationalise what they consider as ‘ethical’ and ‘unethical’ in order to keep functioning in their work contexts [9]. The complexity of today’s workplaces with pressures of externally imposed care and funding pathways recommends that education providers and professional associations need to include ways in which physiotherapists can be supported in situations of discrimination and abuse [38].

The comparison of frequency rankings between WCPT regions demonstrates both different and shared issues in the everyday ethical practice of physiotherapists. The rankings provide a preliminary profile of ethical practice for each world region to inform contextualized codes of ethics, training and support by academic and professional organisations. The rankings also point to the relevance of, and potential for, sharing of resources and action strategies between regions to support global capacity for addressing ethical issues. The approach used recently by the profession to support capacity for local responses to the COVID-19 pandemic via sharing of professional knowledge on facilitated social media network discussions, webinars and an international guidance paper [39] could be similarly applied to the international problem of scarce resources and time affecting quality of physiotherapy treatment. It is acknowledged that the study’s comparative rankings only related to the issues that were listed in the survey. The inclusion of an open question at the end of the survey provided further understanding of what is challenging the ethical practice of physiotherapists in world regions, and its data and analysis are reported in another paper [40].

The study has some limitations. The response rate was low as a calculation of total number of physiotherapists worldwide but compares favourably to an online survey of musculoskeletal physiotherapists offered in 20 different languages which had a response rate of 1307 from 49 countries [41]. It is acknowledged that the experience of physiotherapists in the South America, Africa and North America Caribbean regions was not represented as strongly as other regions in the survey findings. Voices from all regions needs to be heard to inform development of relevant professional training and support. Survey access to physiotherapists in these regions could be increased by offering translated questionnaires and individualising recruitment strategies for each region. The explained variance in the regression analyses are quite low, indicating that other regional, cultural or organisational aspects of diversity not captured by the questionnaire instrument might contribute to the ethical aspects measured. Further qualitative research to explore factors that influence the ethical practice of physiotherapists internationally is needed to determine what other aspects can be included in future quantitative investigations.

## Conclusion

This study provides the first global profile of ethical issues experienced by physiotherapists. Equity in access to, and resources for, physiotherapy is a frequent issue for the profession worldwide. Societal and cultural systems are key influences on the ethical situations that involve physiotherapists in their everyday practice. Interprofessional practice presents frequent challenges to physiotherapists providing ethical and quality care for clients. Working for longer in the profession and having basic ethics education predicts less frequent experience of ethical issues in physiotherapy practice. Physiotherapists globally need support from their workplace organisations, academic institutions and professional associations, and robust ethical training in their education, to assist them to be active moral agents in the complex and pressured workplaces of the twenty-first century.

## Supplementary Information

**Additional file 1: Appendix 1.** Survey questionnaire. Text of questionnaire included in the online survey.

**Additional file 2: Appendix 2.** Variables (units) used in regression analysis. Table showing coding of variables (units) used in regression analyses.

A**Additional file 3: Appendix 3.** Mean age (SD) and number (%) of participants for each characteristic by survey completion versus non-completion. Table showing characteristics of completers and non-completers of the survey.

## Data Availability

The datasets generated and/or analysed during the current study are not publicly available due to containing information that could compromise the privacy of research participants but are available from the corresponding author on reasonable request.

## Abbreviation

WCPT: World Confederation for Physical Therapy associations (now titled ‘World Physiotherapy’)
