# Supplementary material for: Scarcity of resources and inequity in access are frequently reported ethical issues for physiotherapists internationally: an observational study
Source: BMC Med Ethics. 2021 Jul 20;22:97. doi: 10.1186/s12910-021-00663-x (PMC8290210; doi:10.1186/s12910-021-00663-x)
Supplement: Supplementary file 1 — Additional file 1: Appendix 1. Survey questionnaire. Text of questionnaire included in the online survey. [file 12910_2021_663_MOESM1_ESM.docx]

## Appendix 1. Survey questionnaire

## Section 1

## Demographics

## 1. What is your age in years? (We will protect this information when presenting the study-results)

#### 2. What is your nationality? Select all options that apply:

Afghanistan

Albania

Algeria

Andorra

Angola

Antigua and Barbuda

Argentina

Armenia

Australia

Austria

Azerbaijan

Bahamas

Bahrain

Bangladesh

Barbados

Belarus

Belgium

Belize

Benin

Bhutan

Bolivia

Bosnia and Herzegovina

Botswana

Brazil

Brunei

Bulgaria

Burkina Faso

Burundi

Cabo Verde

Cambodia

Cameroon

Canada

Central African Republic (CAR)

Chad

Comoros

Chile

China

Colombia

Democratic Republic of the Congo

Republic of the Congo

Costa Rica

Cote d'Ivoire

Croatia

Cuba

Cyprus

Czech Republic

Denmark

Djibouti

Dominica

Dominican Republic

Ecuador

Egypt

El Salvador

Equatorial Guinea

Eritrea

Estonia

Ethiopia

Fiji

Finland

France

Gabon

Gambia

Georgia

Germany

Ghana

Greece

Grenada

Guatemala

Guinea

Guinea-Bissau

Guyana

Haiti

Honduras

Hungary

Iceland

India

Indonesia

Iran

Iraq

Ireland

Israel

Italy

Jamaica

Japan

Jordan

Kazakhstan

Kenya

Kiribati

Kosovo

Kuwait

Kyrgyzstan

Laos

Latvia

Lebanon

Lesotho

Liberia

Libya

Liechtenstein

Lithuania

Luxembourg

Macedonia (FYROM)

Madagascar

Malawi

Malaysia

Maldives

Mali

Malta

Marshall Islands

Mauritania

Mauritius

Mexico

Micronesia

Moldova

Monaco

Mongolia

Montenegro

Morocco

Mozambique

Myanmar (Burma)

Namibia

Nauru

Nepal

Netherlands

New Zealand

Nicaragua

Niger

Nigeria

North Korea

Norway

Oman

Pakistan

Palau

Palestine

Panama

Papua New Guinea

Paraguay

Peru

Philippines

Poland

Portugal

Qatar

Romania

Russia

Rwanda

Saint Kitts and Nevis

Saint Lucia

Saint Vincent and the Grenadines

Samoa

San Marino

Sao Tome and Principe

Saudi Arabia

Senegal

Seychelles

Sierra Leone

Singapore

Slovakia

Slovenia

Solomon Islands

Somalia

South Africa

South Korea

South Sudan

Spain

Sri Lanka

Sudan

Suriname

Swaziland

Sweden

Switzerland

Syria

Taiwan

Tajikistan

Tanzania

Thailand

Timor-Leste

Togo

Tonga

Trinidad and Tobago

Tunisia

Turkey

Turkmenistan

Tuvalu

Uganda

Ukraine

United Arab Emirates (UAE)

United Kingdom (UK)

United States of America (USA)

Uruguay

Uzbekistan

Vanuatu

Vatican City (Holy See)

Venezuela

Vietnam

Yemen

Zambia

Zimbabwe

Other (please specify)

#### 3. What is your gender?

Female

Male

Other/diverse

#### 4. In which country are you currently working?

(Same answer-possibilities given as in question 2)

#### 5. In which country did you receive your basic Physical Therapy training?

(Same answer-possibilities given as in question 2)

#### 6. In your country of work, who pays for people to have Physical Therapy? Select all options that apply.

Private funding - patient or family/ caregiver (Out of their own pocket)

Private funding - (Insurance, organisation)

Public/ governmental funding

A combination of public/ governmental funding and a private stake

Charities

Other (please specify)

#### 7. How many years have you worked as a Physical Therapist (full time or part time)?

Still in training

Please add exact years:

#### 8. What is the highest educational level you have achieved as a Physical Therapist?

Bachelor/diploma/physiotherapy school

Graduate diploma in physical therapy

Masters degree in physical therapy

Professional doctorate in physical therapy (DPT)

Research doctorate in physical therapy

Bachelor/diploma in another discipline

Graduate diploma in another discipline

Masters degree in another discipline

Professional doctorate in in another discipline

Research doctorate in another discipline

Other (please specify)

#### 9. Which type of workplaces have you worked in as a Physical Therapist? Select all options that apply:

Employed in a private clinic/practice

Employed in a private hospital

Employed in a private rehab

Employed in a government/public clinic/practice

Employed in a government/public hospital

Employed in a government/ public rehab

Employed in a teaching institution

Employed in a research institution

Employed in a sports club

Self-employed

Owner of a clinic/ facility

Other (please specify)

#### 10. What type of areas have you worked in as a Physical Therapist? Select all options that apply:﻿

Rural area

Urban area

#### 11. Which fields of Physical Therapy have you worked in? Select all options that apply:

Acupuncture and Dry Needling

Animal practice

Aquatic therapy

Cardiorespiratory physical therapy

Education in physical therapy

Disability (intellectual and physical)

Health promotion (includes non-communicable/chronic disease management, physical activity)

Information management and technology

Management/administration (includes leadership, medico-legal, professional standards and best practice)

Mental health

Neurology

Occupational health and ergonomics

Oncology/palliative care

Orthopaedics/manual therapy

Older people

Pediatrics

Rehabilitations (includes orthopaedics, neurogical, amputee)

Research in physical therapy

Sports physical therapy

Women’s, Men’s and Pelvic health

Other (please specify)

#### 12. Did you learn about a professional Code of Conduct or Code of Ethics during your basic Physical Therapy Training/ Education?

No

Yes

Don’t know

#### 13. Did you learn about specific ethical decision-making or ethical reasoning frameworks during your Physical Therapy Training/ Education?

No

Yes

Don’t know

If yes, can you name the framework(s)?

## Section 2

## Please answer the following questions to help us understand what ethical situations you experience in your Physical Therapy practice and how frequently you experience them. Select all the ethical situations that you have experienced in your Physical Therapy practice from the following list. For each situation you select, please indicate how frequently you experience or observe it in others on the five-point scale.

## What is an Ethical Situation?

### An ethical situation can be any issue in which an ethical tension is created in the physiotherapist’s practice — for example, a conflict of values, beliefs, or norms; uncertainty as to the appropriate ethical action to take; or distress arising from an inability to act in a way that met the professional’s (or the profession’s) ethical standards.

### These situations are divided into four categories:

### Physical Therapist and Patient Interaction (19 situations)

### Physical Therapist and other Health Professionals (including other Physical Therapists) (10 situations)

### Physical Therapist and the System (5 situations)

### Professional and Economic Ethical Situations (12 situations)

##

## Ethical situations in Physical Therapist and Patient interaction

#### 14. Absence of shared decision-making between patient and physical therapist, e.g. paternalism, not culturally wanted or accepted

| Daily | Weekly | Monthly | Yearly or less | Never |
| --- | --- | --- | --- | --- |
|  |  |  |  |  |

#### 15. Failure to gain informed consent, e.g. cultural differences, cognitive impairment, not attempted

| Daily | Weekly | Monthly | Yearly or less | Never |
| --- | --- | --- | --- | --- |
|  |  |  |  |  |

#### 16. Mismatch/ discrepancy between the patient’s expectations and the physical therapist’s expectations of the therapeutic relationship

| Daily | Weekly | Monthly | Yearly or less | Never |
| --- | --- | --- | --- | --- |
|  |  |  |  |  |

#### 17. Mismatch/ discrepancy between patient’s or family/ caregivers’ wishes and physical therapist’s professional judgement

| Daily | Weekly | Monthly | Yearly or less | Never |
| --- | --- | --- | --- | --- |
|  |  |  |  |  |

#### 18. Patient’s privacy and/or dignity not respected during physical therapy treatment, e.g. not draping appropriately, gossiping about patients

| Daily | Weekly | Monthly | Yearly or less | Never |
| --- | --- | --- | --- | --- |
|  |  |  |  |  |

#### 19. Sexual harassment by patient during treatment

| Daily | Weekly | Monthly | Yearly or less | Never |
| --- | --- | --- | --- | --- |
|  |  |  |  |  |

#### 20. Sexual harassment by physical therapist during treatment

| Daily | Weekly | Monthly | Yearly or less | Never |
| --- | --- | --- | --- | --- |
|  |  |  |  |  |

#### 21. Inappropriate relationship between patient and physical therapist during treatment, e.g. intimate friendship, business partnership

| Daily | Weekly | Monthly | Yearly or less | Never |
| --- | --- | --- | --- | --- |
|  |  |  |  |  |

#### 22. Discrimination by physical therapist towards patient on basis of age, gender, appearance, culture or religion, e.g. refusal to treat, lack of respect for cultural beliefs, poor quality treatment

| Daily | Weekly | Monthly | Yearly or less | Never |
| --- | --- | --- | --- | --- |
|  |  |  |  |  |

#### 23. Physical therapist accepting inappropriate gifts or gratuities

| Daily | Weekly | Monthly | Yearly or less | Never |
| --- | --- | --- | --- | --- |
|  |  |  |  |  |

#### 24. A purposeful absence of truth-telling by patient during treatment

| Daily | Weekly | Monthly | Yearly or less | Never |
| --- | --- | --- | --- | --- |
|  |  |  |  |  |

#### 25. Violence or threatening behaviour by patient towards physical therapist

| Daily | Weekly | Monthly | Yearly or less | Never |
| --- | --- | --- | --- | --- |
|  |  |  |  |  |

#### 26. An absence of purposeful truthtelling by therapist during treatment

| Daily | Weekly | Monthly | Yearly or less | Never |
| --- | --- | --- | --- | --- |
|  |  |  |  |  |

#### 27. Violence or threatening behaviour by physical therapist towards patient

| Daily | Weekly | Monthly | Yearly or less | Never |
| --- | --- | --- | --- | --- |
|  |  |  |  |  |

#### 28. Stopping treatment of a patient when they did not comply with physical therapist’s instruction or advice

| Daily | Weekly | Monthly | Yearly or less | Never |
| --- | --- | --- | --- | --- |
|  |  |  |  |  |

#### 29. Physical therapist prioritising patients for treatment based on reasons other than patient’s clinical need, e.g. cherry picking of easier patients, likelihood of improvement, economic potential

| Daily | Weekly | Monthly | Yearly or less | Never |
| --- | --- | --- | --- | --- |
|  |  |  |  |  |

#### 30. Concerns of the physical therapist regarding treatment of terminally ill patients, e.g. deciding benefit or harm to the patient, futility of treatment, lack of resources

| Daily | Weekly | Monthly | Yearly or less | Never |
| --- | --- | --- | --- | --- |
|  |  |  |  |  |

#### 31. Continuing physical therapy treatment for patient’s psychological/psychosocial support after treatment goals have been reached

| Daily | Weekly | Monthly | Yearly or less | Never |
| --- | --- | --- | --- | --- |
|  |  |  |  |  |

#### 32. Physical therapist abusing their status/power to influence patient’s behavior for their own interest

| Daily | Weekly | Monthly | Yearly or less | Never |
| --- | --- | --- | --- | --- |
|  |  |  |  |  |

## Physical Therapist and other Health Professionals (including other Physical Therapists)

#### 33. Other health professionals seeking financial or other benefit from referring patients to physical therapists

| Daily | Weekly | Monthly | Yearly or less | Never |
| --- | --- | --- | --- | --- |
|  |  |  |  |  |

#### 34. Bullying or harassment of physical therapist by other health professional(s)

| Daily | Weekly | Monthly | Yearly or less | Never |
| --- | --- | --- | --- | --- |
|  |  |  |  |  |

#### 35. Violence or threatening behaviour against patients by other health professionals

| Daily | Weekly | Monthly | Yearly or less | Never |
| --- | --- | --- | --- | --- |
|  |  |  |  |  |

#### 36. Miscommunication, or lack of communication, between physical therapists and other health professionals causing errors and affecting quality in patient care

| Daily | Weekly | Monthly | Yearly or less | Never |
| --- | --- | --- | --- | --- |
|  |  |  |  |  |

#### 37. Physical therapist aware of misconduct by other health professionals, e.g incompetency, violating laws and professional obligations

| Daily | Weekly | Monthly | Yearly or less | Never |
| --- | --- | --- | --- | --- |
|  |  |  |  |  |

#### 38. Prescription and ongoing provision of analgesics and/or sedatives to patients without appropriate review over time

| Daily | Weekly | Monthly | Yearly or less | Never |
| --- | --- | --- | --- | --- |
|  |  |  |  |  |

#### 39. Referrals, or absence of referrals, from other health professionals that constrain the quality of physical therapy services

| Daily | Weekly | Monthly | Yearly or less | Never |
| --- | --- | --- | --- | --- |
|  |  |  |  |  |

#### 40. Respecting the patient's therapeutic relationship with other health professionals, when the physical therapist disagrees with the other health professional's opinion

| Daily | Weekly | Monthly | Yearly or less | Never |
| --- | --- | --- | --- | --- |
|  |  |  |  |  |

#### 41. Conflict with another health professional about patient’s management

| Daily | Weekly | Monthly | Yearly or less | Never |
| --- | --- | --- | --- | --- |
|  |  |  |  |  |

#### 42. Inappropriate, insulting or offending behavior among colleagues on social media

| Daily | Weekly | Monthly | Yearly or less | Never |
| --- | --- | --- | --- | --- |
|  |  |  |  |  |

## Physical Therapist and the System he or she is working in

#### 43. Physical therapist required by an organisation or system to discharge patient from treatment based on reasons other than patient’s clinical need, e.g. insurance limits, health care system policy

| Daily | Weekly | Monthly | Yearly or less | Never |
| --- | --- | --- | --- | --- |
|  |  |  |  |  |

#### 44. Physical therapist prevented by an organisation or system from treating patient based on clinical need, e.g. health insurance will not cover condition, health care system policy does not allow

| Daily | Weekly | Monthly | Yearly or less | Never |
| --- | --- | --- | --- | --- |
|  |  |  |  |  |

#### 45. Scarce resources and time affecting quality of physical therapy treatment

| Daily | Weekly | Monthly | Yearly or less | Never |
| --- | --- | --- | --- | --- |
|  |  |  |  |  |

#### 46. Physical therapist pressured by organisation or system to return patient to sport or work commitments too early

| Daily | Weekly | Monthly | Yearly or less | Never |
| --- | --- | --- | --- | --- |
|  |  |  |  |  |

#### 47. Withholding or limiting physical therapy services to improve work conditions of the physical therapy provider or for the convenience of the physical therapist, e.g. time, location

| Daily | Weekly | Monthly | Yearly or less | Never |
| --- | --- | --- | --- | --- |
|  |  |  |  |  |

## Professional and Economic ethical situations

#### 48. Breach of patient confidentiality by physical therapist

| Daily | Weekly | Monthly | Yearly or less | Never |
| --- | --- | --- | --- | --- |
|  |  |  |  |  |

#### 49. Fraudulent billing for physical therapy services

| Daily | Weekly | Monthly | Yearly or less | Never |
| --- | --- | --- | --- | --- |
|  |  |  |  |  |

#### 50. Overcharging patients for physical therapy services

| Daily | Weekly | Monthly | Yearly or less | Never |
| --- | --- | --- | --- | --- |
|  |  |  |  |  |

#### 51. Inadequate/ unlawful record keeping by physical therapist

| Daily | Weekly | Monthly | Yearly or less | Never |
| --- | --- | --- | --- | --- |
|  |  |  |  |  |

#### 52. Lack of evidence available to support effectiveness and safety of physical therapy practices

| Daily | Weekly | Monthly | Yearly or less | Never |
| --- | --- | --- | --- | --- |
|  |  |  |  |  |

#### 53. Physical therapist practicing outside of personal scope of knowledge and skills

| Daily | Weekly | Monthly | Yearly or less | Never |
| --- | --- | --- | --- | --- |
|  |  |  |  |  |

#### 54. Physical therapist overtreating patients for own economic gain

| Daily | Weekly | Monthly | Yearly or less | Never |
| --- | --- | --- | --- | --- |
|  |  |  |  |  |

#### 55. Conflict between physical therapist’s professional obligations (as per code of ethics) and cultural or personal values

| Daily | Weekly | Monthly | Yearly or less | Never |
| --- | --- | --- | --- | --- |
|  |  |  |  |  |

#### 56. Physical therapy not accessible to all people in society who need it, e.g. due to cost, lack of services in regions, or discrimination by health care system

| Daily | Weekly | Monthly | Yearly or less | Never |
| --- | --- | --- | --- | --- |
|  |  |  |  |  |

#### 57. Conflict in duties toward employer, third-party payer, and the patient

| Daily | Weekly | Monthly | Yearly or less | Never |
| --- | --- | --- | --- | --- |
|  |  |  |  |  |

#### 58. A lack of advocacy for patient’s interests, needs or supports when they are unable to advocate for themselves

| Daily | Weekly | Monthly | Yearly or less | Never |
| --- | --- | --- | --- | --- |
|  |  |  |  |  |

#### 59. Physical therapist recommending and selling products for own economic gain

| Daily | Weekly | Monthly | Yearly or less | Never |
| --- | --- | --- | --- | --- |
|  |  |  | | |

## Section 3

### Now there is an opportunity to describe an ethical situation that you have experienced which was not on the list. Please briefly describe what you experienced.

60. Please describe an ethical situation you have experienced which was not on the list
