# Supplementary material for: Scarcity of resources and inequity in access are frequently reported ethical issues for physiotherapists internationally: an observational study
Source: BMC Med Ethics. 2021 Jul 20;22:97. doi: 10.1186/s12910-021-00663-x (PMC8290210; doi:10.1186/s12910-021-00663-x)
Supplement: Supplementary file 2 — Additional file 2: Appendix 2. Variables (units) used in regression analysis. Table showing coding of variables (units) used in regression analyses. [file 12910_2021_663_MOESM2_ESM.docx]

Appendix 2. Variables (units) used in regression analysis.

| **Nominal variable** | **Dummy variable** | **Categories** |
| --- | --- | --- |
| Biographical | Gender | Female (0 or 1)  Male (0 or 1)  Diverse (0 or 1) |
|  | WCPT membership (0 or 1) |  |
|  | WCPT region | Africa (0 or 1)  Asia Western Pacific (0 or 1)  Europe (0 or 1)  North America Caribbean (0 or 1)  South America (0 or 1) |
| Educational | Education in physiotherapy (0 or 1) |  |
|  | Learned about codes of conduct/ethics | Yes (0 or 1)  No (0 or 1)  Don’t know (0 or 1) |
|  | Learned about specific ethical reasoning/decision making | Yes (0 or 1)  No (0 or 1)  Don’t know (0 or 1) |
| Vocational | Area where workplace located | Rural (0 or 1)  Urban (0 or 1)  Both (0 or 1) |
|  | Years worked as physiotherapist (years) |  |
|  | Field of physiotherapy practice (sum of each participant’s fields of practice) |  |
|  | Type of workplace (sum of workplaces each participant has worked in) |  |
|  | Paying sources (sum of each participant’s paying sources) |  |
