# Supplementary material for: Scarcity of resources and inequity in access are frequently reported ethical issues for physiotherapists internationally: an observational study
Source: BMC Med Ethics. 2021 Jul 20;22:97. doi: 10.1186/s12910-021-00663-x (PMC8290210; doi:10.1186/s12910-021-00663-x)
Supplement: Supplementary file 3 — AAdditional file 3: Appendix 3. Mean age (SD) and number (%) of participants for each characteristic by survey completion versus non-completion. Table showing characteristics of completers and non-completers of the survey. [file 12910_2021_663_MOESM3_ESM.docx]

Appendix 3. Mean age (SD) and number (%) of participants for each characteristic by survey completion vs. non-completion.

| Characteristic | Completed survey  n=846 | Did not complete survey  n=366 |
| --- | --- | --- |
| Age, mean (SD) | 36.95 (11.60) | 31.35 (11.25) |
| Gender |  |  |
| Female | 560 (66) | 254 (69) |
| Male | 280 (33) | 108 (30) |
| Diverse | 4 (<1) | 4 (1) |
| In Training | 122 (14) | 142 (39) |
| Working | 723 (85) | 224 (61) |
| Geographic region |  |  |
| Africa Region | 101 (12) | 40 (11) |
| Asia Western Pacific Region | 252 (30) | 132 (36) |
| Europe Region | 378 (45) | 156 (43) |
| North America Caribbean Region | 106 (13) | 32 (9) |
| South America Region | 9 (1) | 6 (2) |
| Type of Workplace |  |  |
| Private | 829 (98) | 274 (75) |
| Government/Public | 684 (81) | 207 (57) |
| Teaching Institution | 218 (26) | 58 (16) |
| Research Institution | 78 (9) | 18 (5) |
| Sports Club | 97 (11) | 45 (12) |
| Self Employed/Owner | 335 (40) | 120 (33) |
| Other | 88 (10) | 52 (14) |
| Area where workplace located |  |  |
| Rural Area | 82 (10) | 38 (10) |
| Urban Area | 503 (59) | 239 (65) |
| Both Areas | 261 (31) | 85 (23) |
| Paying sources |  |  |
| Private Funding (patient or family) | 473 (56) | 209 (57) |
| Private Funding (organization) | 416 (49) | 141 (39) |
| Public/Governmental Funding | 405 (48) | 144 (39) |
| Combination of Public/Governmental funding and Private | 439 (52) | 170 (46) |
| Charities | 107 (13) | 46 (13) |
| Other | 23 (3) | 7 (2) |
| Field of physiotherapy practice |  |  |
| Acupuncture, Dry Needling | 128 (15) | 42 (11) |
| Animal | 10 (1) | 3 (1) |
| Aquatic | 127 (15) | 51 (14) |
| Cardiorespiratory | 266 (31) | 110 (30) |
| Education | 254 (30) | 95 (26) |
| Disability | 207 (24) | 82 (22) |
| Health Promotion | 213 (25) | 87 (24) |
| Information Management | 25 (3) | 13 (4) |
| Management/Administration | 153 (18) | 41 (11) |
| Mental Health | 68 (8) | 29 (8) |
| Neurology | 367 (43) | 126 (34) |
| Occupational Health/Ergonomics | 133 (16) | 40 (11) |
| Oncology/Palliative Care | 130 (15) | 33 (9) |
| Orthopaedics/Manual Therapy | 552 (65) | 185 (51) |
| Older People | 393 (46) | 127 (35) |
| Paediatrics | 239 (28) | 100 (27) |
| Rehabilitation | 539 (64) | 185 (51) |
| Research | 164 (19) | 52 (14) |
| Sport | 303 (36) | 116 (32) |
| Women’s, Men’s and Pelvic Health | 155 (18) | 60 (16) |
| Other | 81 (10) | 44 (12) |
| Highest educational level achieved (in physiotherapy or other discipline) |  |  |
| Bachelor/diploma | 456 (54) | 207 (57) |
| Graduate diploma | 101 (12) | 53 (14) |
| Masters degree | 294 (35) | 73 (20) |
| Professional doctorate | 92 (11) | 37 (10) |
| Research doctorate | 50 (6) | 14 (4) |
| Other | 72 (9) | 43 (12) |
| Learned about code of conduct/ethics during basic physiotherapy education |  |  |
| Yes | 637 (75) | 256 (70) |
| No | 147 (17) | 71 (20) |
| Don’t Know | 61 (7) | 38 (10) |
| Learned about specific ethical decision-making/reasoning during basic physiotherapy education |  |  |
| Yes | 409 (48) | 174 (48) |
| No | 292 (35) | 107 (29) |
| Don’t Know | 145 (17) | 84 (23) |
